# High Gain Flat-Panel mmWave Antenna Array

**DOI:** 10.3390/s23239433

**Published:** 2023-11-27

**Authors:** Seong-Mo Moon, Junhyuk Cho, Han Lim Lee

**Affiliations:** 1 Satellite Payload Research Section, ETRI, Daejeon 34129, Republic of Korea; smmoon@etri.re.kr; 2 School of Electrical and Electronics Engineering, Chung-Ang University, Seoul 06974, Republic of Korea; ny845@cau.ac.kr

**Keywords:** flat-panel antenna array, high gain antenna, mmWave antenna, mmWave antenna array

## Abstract

In the realm of mmWave communication and connectivity, integrating chips and antennas into a cohesive system is paramount. Given this, planar antenna arrays have become indispensable. In this article, we introduce a novel antenna array tailored for mmWave applications, characterized by its high directivity. Distinctively, this new array employs a flat-panel radiator, ensuring an augmented gain without necessitating additional superstrate layers. To validate its potency, a 4 × 4 flat-panel array with dimensions of 3.74 λ_0_ × 3.74 λ_0_ × 0.106 λ_0_ at 28 GHz including a ground plane was designed and tested for n257 band. The standalone array element exhibited a bandwidth of 20.6%, centered at 28.5 GHz. Furthermore, a 1 × 16 mmWave feed network was designed and amalgamated with the array elements to assess the comprehensive antenna performance. The measured peak gain of 21.3 dBi at 28.5 GHz was observed with the measured half power beamwidth of 15° while the gain variation within the operation band was less than 3 dB.

## 1. Introduction

**Figure 1:**
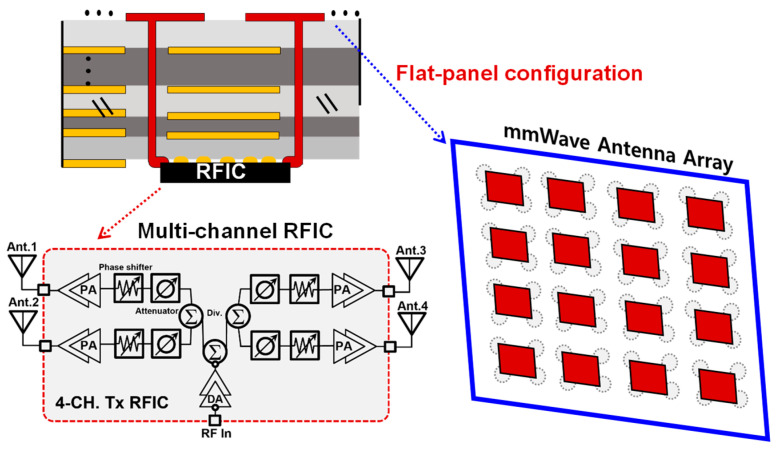
Flat-panel antenna array configuration for improving integration efficiency with RFICs.

The advent of 5G communication heralds a fervent era of refinement in mmWave antenna technology, strategically positioned to capture the untapped commercial landscape. The peculiarities of mmWave, such as heightened propagation loss and linear trajectory, necessitate the incorporation of beam-forming technology complemented by high-directivity antennas, an essential advancement for the imminent 5G evolution and its successors, B5G and 6G. The seamless fusion of cutting-edge beam-forming technology into forthcoming mmWave applications mandates the effective amalgamation of array antennas with single or multiple beam-forming RFICs. This integration signifies not merely an elevation in electrical prowess but also a simplification of physical manufacturability. Figure 1 delineates the optimal physical and electrical attributes desired for a mmWave antenna module. At the mmWave frontier, low-profile or planar-style antenna components are coveted, courtesy of their compatibility with multi-layered printed circuit boards (PCBs) or their amenability to sophisticated fabrication methodologies. To thwart performance diminution at high frequencies—often the consequence of parasitic capacitances, inductive hurdles, and dielectric losses—the array antenna and RFIC ought to be coalesced in both a compact and planar fashion [1]. For instance, mitigating additional feed line losses from RFICs to antenna components is feasible by the direct interfusion of beamforming TRx channels with antenna elements, be it on identical PCBs or within an advanced package framework. This symbiotic antenna-RFIC entity could then function as a modular component, readily scalable for more extensive array systems. Moreover, the uncharted complexities of B5G hardware, particularly the amplification in array antenna elements to counteract pronounced attenuation in mmWave, demand comprehensive contemplation. The escalation in antenna constituents inherent in beam-forming approaches correlates with a burgeoned RF system load, encompassing beam-forming ICs. Thus, a delicate equilibrium of system efficacy and cost-efficiency becomes paramount. A viable resolution emerges in the guise of high-gain radiating components for the beam-forming antenna module, as illustrated in Figure 1. Should a high-directivity antenna succeed in generating the requisite gain with fewer elements relative to extant patch-type counterparts, the necessity for RF conduits or RFICs could diminish. In essence, improving the gain beyond the conventional mmWave planar patch antennas, typically registering at 7 or 8 dBi [2,3], could significantly bolster overall system efficiency.

Past investigations have detailed the use of various configurations to bolster antenna gain. Arrays of printed dipoles with series-fed networks, and log-periodic antennas with supplementary directors [4,5,6] have been investigated, yet these techniques often require increased planar space without proportionately boosting gain compared to standard patches. Additionally, these configurations rely on end-fire radiation, which does not facilitate the integration of two-dimensional planar phased arrays with multiple multi-channel RFICs. Substrate-integrated waveguide (SIW) antennas are another area that has received attention for high-gain mmWave applications [7,8,9,10,11,12,13]. However, the necessity of extra layers to construct cavities and the precision required for via fabrication, together with the inherent dielectric losses in SIW feed networks, significantly impact radiation efficiency. This makes SIW antennas impractical for large-scale phased array implementations. These studies underscore the imperative to surmount the electrical and physical production challenges posed by existing designs to advance the state of antenna technology. High-gain alternatives for mmWave bands have included the use of Luneburg lens and multi-layered superstrate designs [14,15,16]. Despite their potential, these solutions face challenges, including bulkiness, complex integration, limited steering capabilities, and variable gain across operational frequencies, rendering them suboptimal for phased array systems. Dielectric resonator antennas have also been considered for high gain mmWave solutions. However, the substantial structure and complex fabrication processes pose significant barriers to their practical deployment. To address these issues, a novel mmWave planar high gain antenna design has been introduced, which does not rely on additional superstrates or cavities [17]. However, this antenna has only been tested as a singular element, even though mmWave antennas are predominantly designed in array configurations. Given the significance of the coupling effect in mmWave antenna array design, it is essential to examine the adaptability of the previously reported element when expanded into an array. Consequently, this article presents the development of the high gain planar radiator into a 4 × 4 array, complete with a feed network, tailored for the n257 operation band. In brief, the contribution of this article can be summarized as follows.

- An extended analysis of a high-gain antenna element [17] for an array antenna application.
- The design and verification of a feed network tailored for the high-gain antenna array, implemented with a 4 × 4 array antenna.
- The development and validation of an optimized 4 × 4 high gain antenna array tailored for the n257 band.

## 2. Design and Analysis

**Figure 2:**
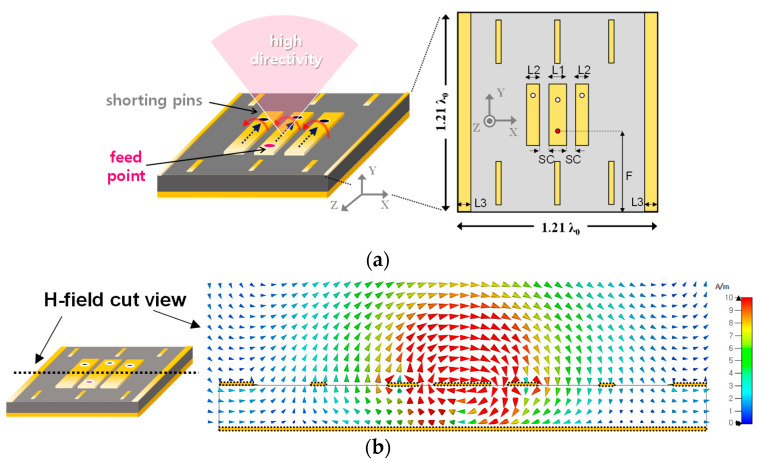

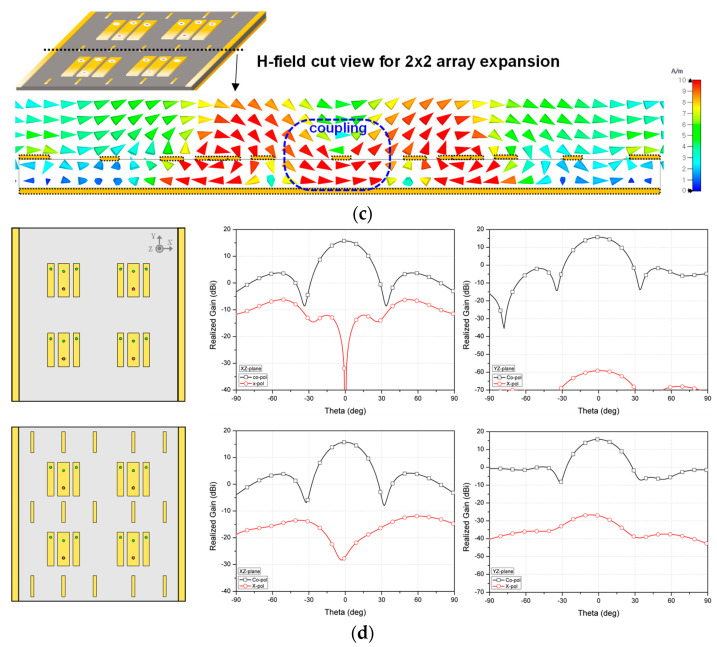
Overview of (**a**) previously reported high gain antenna (L1 = 1.5 mm, L2 = 0.8 mm, L3 = 1 mm, SC = 0.45 mm, F = 5.4 mm) [17], (**b**) simulated magnetic field, (**c**) simulated magnetic field in the 2 × 2 array, and (**d**) simulated gain comparison between different 2 × 2 configurations.

Figure 2a illustrates the previously reported antenna structure [17] to be referenced in this article. Instead of the traditional single flat conductor seen in standard patch antennas, this structure utilizes multiple segmented planar conductors connected by shorting pins. This design increases the field apertures and aligns the phase of the resonant currents across each conductor segment. This results in a cumulative effect, enhancing the antenna’s directivity. When a signal is introduced via the lone signal port, surface currents generate magnetic fields along each conductor. The spaces between these conductors offer additional routes for the circulating field. The shorting pins on each conductor segment ensure the phase synchronization of the surface currents. This synchronization allows for the overlapping of magnetic fields, leading to an increase in gain due to field synthesis as shown in Figure 2b. Since the detailed analysis for the single element can be found in [17], the detailed design steps will be omitted in this article. However, when it is expanded into an array, additional conductors placed amid multiple elements appear to be non-contributive, as shown in Figure 2c. Contrary to the single element radiation, the array’s magnetic field seems to link with adjacent edge conductors rather than looping around each conductor individually. This suggests that the supplementary conductors cannot improve a beneficial antenna gain in the array. The detailed geometric parameters and performance of the single element can be found in [17] as a reference. To further illustrate the array performance, two 2 × 2 models, based on the elements described in [17], were simulated to assess the realized gain in both E- and H-planes, as depicted in Figure 2d. While the single element incorporating extra conductors exhibited a higher gain compared to the variant without extra conductors, the realized gain in the array configurations did not demonstrate an enhancement in gain.

**Figure 3:**
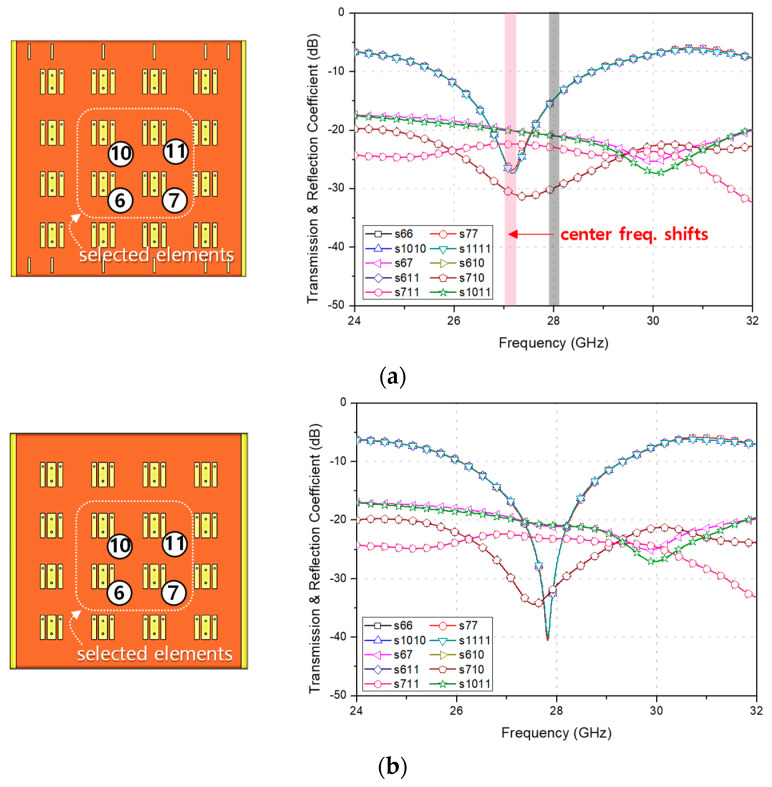
Simulated array designs featuring selected transmission and reflection coefficients for (**a**) the preliminary 4 × 4 array incorporating additional parasitic conductors and (**b**) the refined 4 × 4 array layout.

The direct conversion of the previously detailed element into an array layout has not enhanced the antenna gain, indicating a need for further array optimization. Figure 3a shows a simulation of the 4 × 4 array, featuring antenna spacing of 0.457 λ_0_ and a ground plane dimension of 3.74 λ_0_ × 3.74 λ_0_ at 28 GHz. Notably, the intermediary parasitic conductors, which hamper antenna gain, have been removed, retaining only the peripheral parasitic conductors. Subsequently, four central elements are selected to represent standard reflection and antenna coupling characteristics. However, the plot in Figure 3a demonstrates a shift in the center frequency, attributed to the residual peripheral parasitic conductors. To address this, these extra conductors were discarded, and the antenna’s edge-to-edge spacing is fine-tuned to 0.438 λ_0_. As a result, the simulations, depicted in Figure 3b, meet the n257 band requirements, achieving a 10-dB impedance bandwidth of 13.7% centered at 28 GHz.

**Figure 4:**
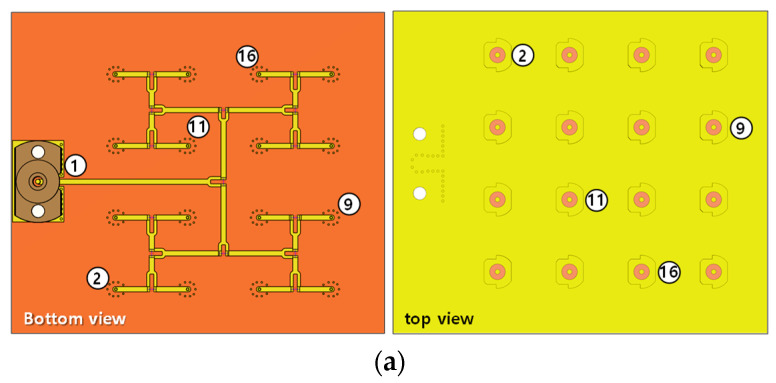

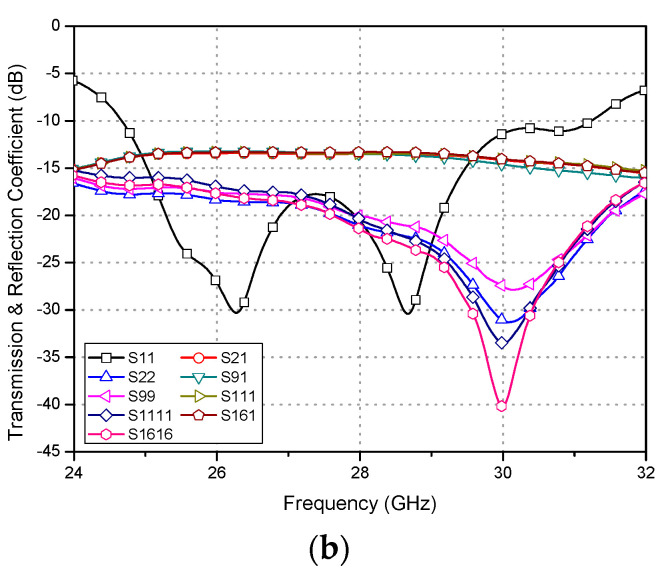
1 × 16 feed network with (**a**) designed structure showing selected test ports, and (**b**) simulated transmission and reflection coefficients.

Next, a 1 × 16 feed network based on the Wilkinson divider is designed to feed the 16 antenna elements, as illustrated in Figure 4a. The 1 × 16 Wilkinson divider is crafted using a substrate with a relative permittivity of 3.55 and a thickness of 0.305 mm. Bearing real-world applications in multilayered configurations in mind, an extra substrate layer with 0.508 mm thickness is additionally inserted to enhance the rigidity of the printed circuit board (PCB). This results in the feed network being structured on a four-layer PCB. Additionally, to minimize the transition loss from the microstrip line to signal via, grounded-guide vias are positioned surrounding the signal vias for every output port. For clarity in presenting simulated outcomes, four ports from each subsection of the feed network have been selected, as shown in Figure 4a.

**Figure 5:**
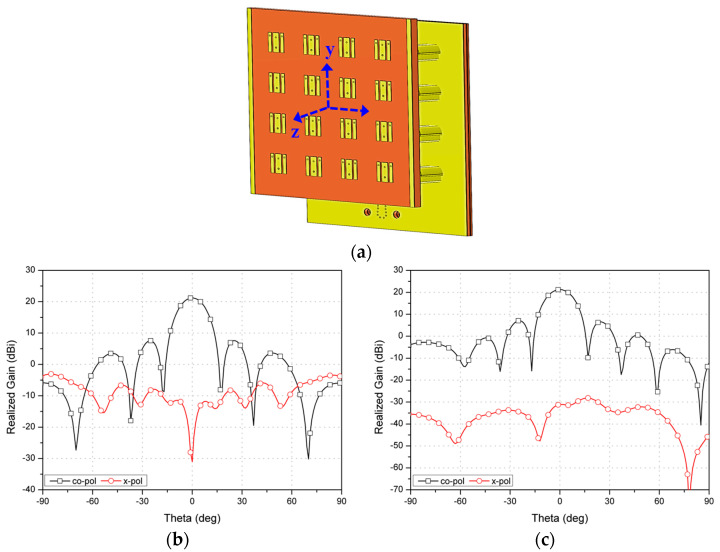
Proposed 4 × 4 SPA array with feed network: (**a**) 3-D simulation model and radiation patterns in (**b**) xz-plane and (**c**) yz-plane at 28 GHz.

Subsequently, Figure 4b depicts the simulated reflection and transmission coefficients. Within the simulation range of 24 GHz to 32 GHz, the 10-dB impedance bandwidth for port 1 is approximately 25.1%, with the remaining ports more than 28%. Moreover, the peak transmission loss as gauged through the feed network is roughly 1.5 dB within the active bandwidth of 26.5 GHz to 29.5 GHz. Then, the 4 × 4 flat panel array is combined with the 1 × 16 feed network to simulate the array radiation patterns, as shown in Figure 5a. The type-B array shows the simulated peak gain of 21.2 dBi at 28 GHz in both xz and yz planes. The simulated HPBW in xz and yz planes are about 15.1° and 14.6°, respectively. The simulated side lobe level (SLL) is always lower than −13 dB and the minimum cross-polarization discrimination (XPD) level within the HPBW is always better than 20 dB.

## 3. Fabrication and Measurement

**Figure 6:**
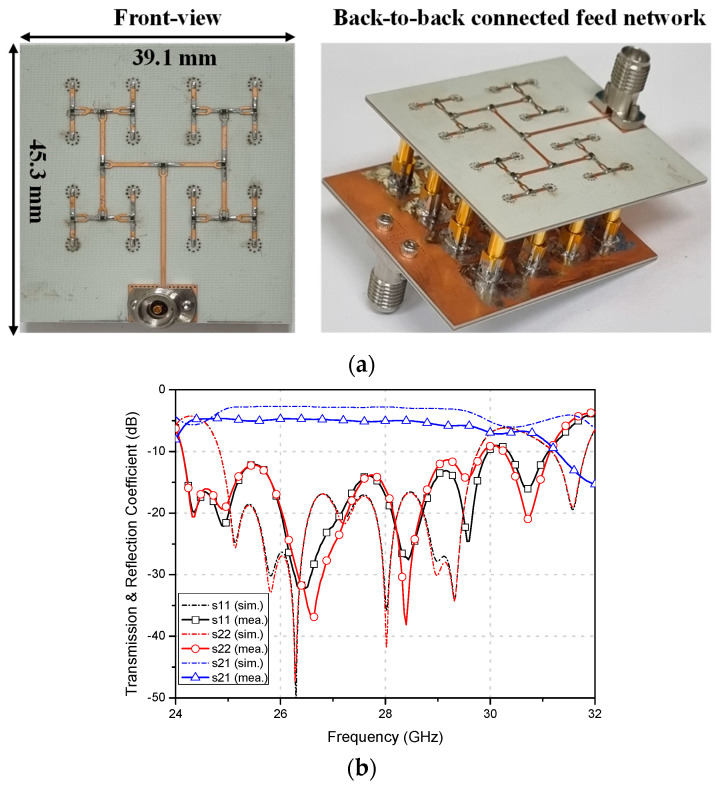
Fabricated 1 × 16 array feed network with (**a**) back-to-back connection and (**b**) measured transmission and reflection coefficients.

To verify the proposed flat-panel antenna array performances, the 1 × 16 feed network was first fabricated with a Rogers RO4003C substrate having a relative permittivity of 3.55, a loss tangent of 0.027, and a thickness of 0.508 mm, as described in the previous section. Also, an additional RO4003C with a thickness of 0.305 mm was additively fabricated for PCB rigidity. Here, RO4450T with a relative permittivity of 3.35 was used as a prepreg. The fabricated feed network is shown in Figure 6a with a back-to-back connection for measurement. The measured 10-dB impedance bandwidth of the feed network was more than 25% and the measured path loss at 28 GHz was about 2.5 dB as shown in Figure 6b.

**Figure 7:**
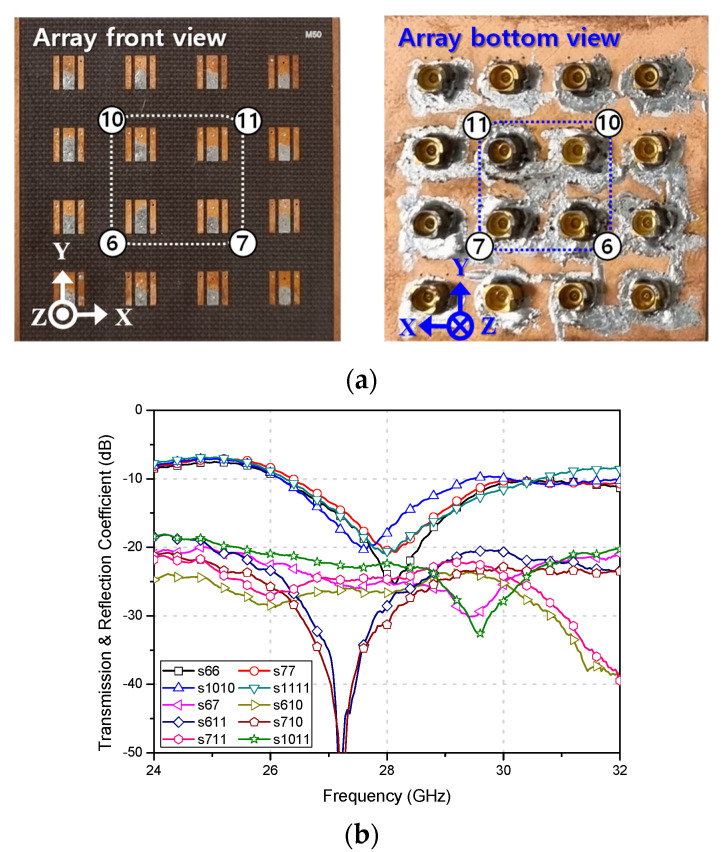
Proposed flat-panel antenna array with (**a**) fabrication (**b**) measured transmission and reflection coefficients.

Next, the proposed 4 × 4 flat-panel antenna array was fabricated using the TLX-9 substrate, with a ground size of 40.1 mm × 40.1 mm, as depicted in Figure 7a. To assess the inherent characteristics of the antenna element within the array and subsequently connect the array board to the 1 × 16 feed network, 16 SMPM RF connectors were employed. Additionally, four ports located centrally in the array, which experience the most significant coupling effect, were chosen to represent the array elements’ transmission and reflection coefficients. As per Figure 7b, the measured maximum 10-dB impedance bandwidth was 20.6%, and the minimum isolation at 28 GHz exceeded 20.5 dB.

## 4. Discussion

**Figure 8:**
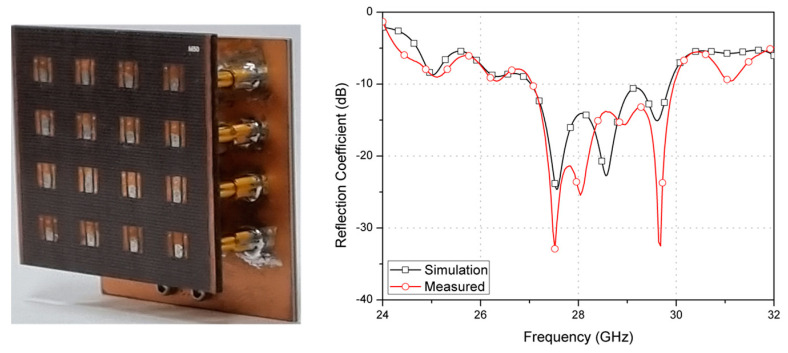
Proposed flat-panel antenna array with feed network and measured reflection coefficients.

The array antenna and feed network were integrated, as depicted in Figure 8. The measured 10-dB impedance bandwidth of the array with the integrated feed network was 10.4%, meeting the target operation band from 26.5 GHz to 29.5 GHz. Both the simulated and measured results are in good agreement.

**Figure 9:**
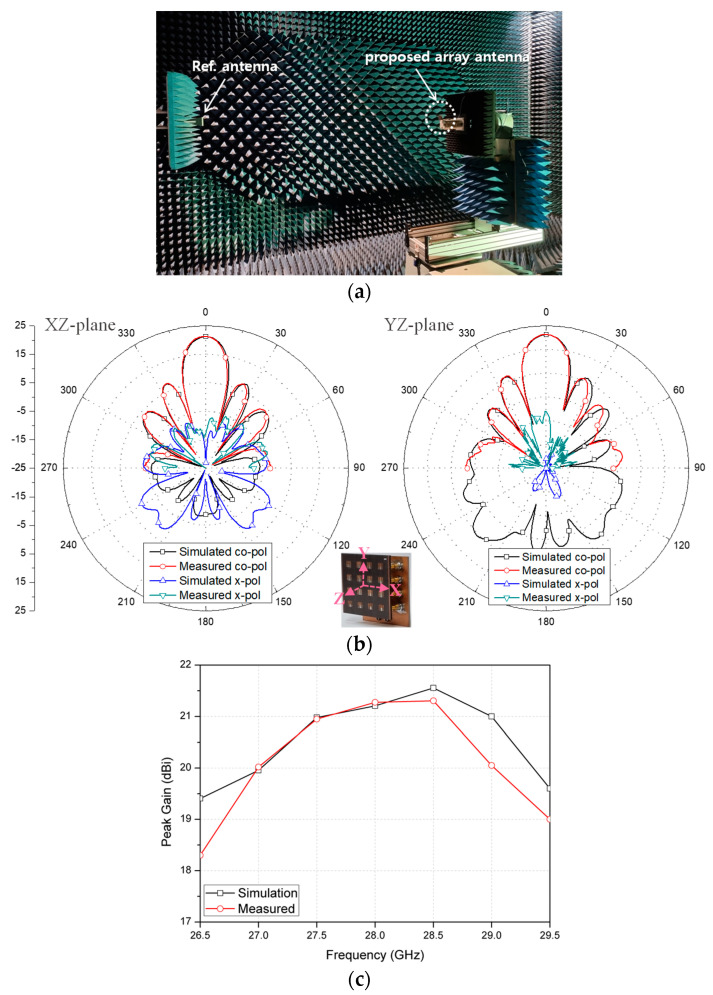
Simulated and measured radiation patterns of the proposed flat-panel array with feed network: (**a**) measurement at mmWave anechoic chamber, (**b**) radiation pattern and (**c**) peak gain variation within the operation band.

Then, the radiation patterns of the proposed flat-panel array with the feed network were measured at 28 GHz as shown in Figure 9a. The measured peak gains in xz and yz planes of the proposed flat-panel antenna array were about 21.2 dBi and 21.3 dBi, respectively, with SLL less than −13.5 dB. Also, the measured HPBWs in xz and yz planes were 15° and 14.3°, respectively. The measured XPD levels were better than 21. 6 dB. Further, Figure 9b shows the simulated and measured peak gains from 26.5 GHz to 29.5 GHz. Within the operation band, the proposed flat-panel antenna array showed the maximum gain of 21.3 dBi at 28.5 GHz while the measured gain variations over the operation band was less than 3 dB.

Lastly, the proposed flat-panel antenna array is compared with other cutting-edge mmWave antenna arrays, as detailed in Table 1. Two figure-of-merits (FoMs) were employed for the antenna bandwidth per substrate thickness and antenna gain per array area to compare with the other antennas. It is important to highlight that the presence of dummy layers in our proposed antenna led to a marginally reduced FoM. Nevertheless, the flat-panel array demonstrated superior gain combined with an expansive bandwidth compared to other array with a similar FoM, underscoring its aptitude for mmWave applications.

## 5. Conclusions

In this article, we expanded the previously discussed planar segmented antenna element into the 4 × 4 configuration, integrating the 1 × 16 feed network. Both the individual antenna array and the feed network were first tested separately, then combined to verify the overall performance. The resulting 4 × 4 flat-panel antenna array displayed a peak measured gain of 21.3 dBi with the total volume of 3.74 λ_0_ × 3.74 λ_0_ × 0.106 λ_0_, including the ground plane, at 28.5 GHz. The measured array gain variation remained below 3 dB within the operational band. The proposed array’s ability to improve mmWave antenna directivity with fewer components, coupled with its flat-panel geometry facilitating integration with RFICs, holds significant potential for future mmWave applications. Consequently, although this article has verified the pure performance of the antenna, comprehensive testing of the complete antenna array, including RFIC integration, is identified as an area for future work.

## Figures and Tables

**Table 1:** Comparison of the proposed flat-panel array with other state-of-the-art arrays.

Ref.	Center Freq. (GHz)	# of Antenna Elements	Total Antenna Volume $(\lambda_{0}^{3}$ )	Bandwidth (%)	Realized Gain (dBi)	FoM 1 (BW/ $\lambda_{0}$ **)	FoM 2 (Gain/ $\lambda_{0}^{2}$ ***)
[18]	30	16 (array)	3.5 × 3.5 × 0.28	16.5	21.2	17.678	1.73
[19]	27.7	42 (array)	6.88 × 8.27 × 0.047	6.3	21.8	37.129	0.383
[20]	28	16 (array)	3.92 × 3.55 × 0.093	8.5	19.1	25.591	1.372
[21]	30	16 (array)	5.7 × 5.7 × 0.152	16.7	19.3	32.96	0.594
[22]	30	16 (array)	3.72 × 3.72 × 0.12	11.89	17.37	29.725	1.255
[23]	28	16 (array)	7.34 × 2.1 × 0.047	4.28	18.2	25.532	1.18
[24]	28	25 (array)	4.00 × 4.67 × 0.146	52	20.44	99.726	1.094
This work	28.5	16 (array)	3.74 × 3.74 × 0.106 *	20.6	21.3 *	55.386	1.522

* Excluding the feed network fabricated separately as displayed in Figure 6. ** Antenna board thickness. *** Array antenna area.

## Data Availability

Data are contained within the article.
